# New, potent, small molecule agonists of tyrosine kinase receptors attenuate dry eye disease

**DOI:** 10.3389/fmed.2022.937142

**Published:** 2022-08-25

**Authors:** Zhiyuan Yu, Shaon Joy, Tianxiong Mi, Ghasem Yazdanpanah, Kevin Burgess, Cintia S. de Paiva

**Affiliations:** ^1^Department of Ophthalmology, Ocular Surface Center, Baylor College of Medicine, Cullen Eye Institute, Houston, TX, United States; ^2^Department of Chemistry, Texas A&M University, College Station, TX, United States

**Keywords:** dry eye, tyrosine kinase receptors agonists, goblet cell, NFkapapB, inflammation, gene expression, *Ptger*, prostaglandin E receptor

## Abstract

Nerve growth factor (NGF), brain-derived neurotrophic factor (BDNF), and neurotrophin3 (NT-3) bind to tyrosine kinase (Trk) receptors, TrkA, TrkB, and TrkC, respectively. This study investigated the efficacy of novel molecule agonists of Trk receptors in an *in vivo* model of dry eye disease (DED). Small molecule TrkC agonist (C1) and a pan-Trk agonist (pan) were synthesized for this. C57BL/6J mice were subjected to desiccating stress (DS) and received bilateral eye drops of **C1**, **pan,** or vehicle (2x/day). Dry eye signs, inflammation and expression of corneal barrier function, and conjunctival goblet cell (GC) densities were measured as part of the DED phenotype. Corneal epithelial lysates were collected for either western blot or RNA extraction. Extracted total RNAs were used for NanoString analyses. Immunofluorescent staining was performed on whole-mount corneas using anti-TNFAIP3 and anti-EP4 antibodies. Compared to vehicle, mice subjected to desiccating stress and treated with agonists **pan** and **C1** showed improved corneal barrier function, while **C1** also increased GC density. NanoString analyses revealed upregulation of specific mRNA transcripts (*Ptger4, Tnfaip3*, *Il1a and Ptger4*, *Tlr3*, *Osal1)* in **pan-** and **C1**-treated corneas compared to vehicle-treated corneas. Western blots showed that **pan** and **C1** decreased vehicle-induced NFkB nuclear translocation after DS for one day and increased EP4 and TNFAIP3 protein levels after 5 days of DS in corneal epithelium lysates. We conclude that small-molecule agonists of Trk receptors improve DED by decreasing NFkB activation and increasing protein expression of anti-inflammatory molecules TNFAIP3 and EP4. Surprisingly, the most efficacious small molecule agonists were not TrkA selective but TrkC and panTrk, suggesting that wider exploration of TrkB and C and pan Trk agonists are warranted in efforts to treat DED.

## Introduction

Dry eye disease (DED) affects millions of patients worldwide. Clinically, patients complain of irritation, burning, and pain. Dry eye disease is characterized by decreased corneal sensitivity and cornea epitheliopathy; in severe cases, these can lead to ocular perforations. Irregular corneal surfaces caused by DED can lead to loss of functional vision and loss, significantly impacting daily activities, such as driving, reading, and using a computer.

Neurotrophins, protein growth factors, including nerve growth factor (NGF), brain-derived neurotrophic factor (BDNF), neurotrophin3 (NT-3), and NT-4, regulate the growth, survival, and differentiation of neurons and many other neuroectoderm tissues (1). Most “positive” signaling from neurotrophins is mediated by binding to tyrosine kinase (Trk) receptors. These Trk receptors are selective (NGF for TrkA; BDNF and NT-4 for TrkB; and NT-3 for TrkC) but not specific. For instance, NT-3 binds TrkA with a lower affinity than TrkC.

Neurotrophins, NGF, NT-3, –4, BDNF, and all the Trk receptors, are in the stroma and epithelial cells of the cornea (2). Breakdown of nerve distribution in the cornea causes epithelium disruption and ulceration (3). Corneal nerves (and other regions around the eye) secrete growth factors like these to counteract epithelium disruption. Hence, neurotrophins are important for corneal function. Corticosteroids can interact with the receptor tyrosine kinase for BDNF (TrkB), playing an important role in the beneficial effects of BDNF. As such, corticosteroids can be useful in DED therapy (4).

Neurotrophins are intimately related to wound healing in the cornea, but the potential of small molecules which selectively stimulate Trk receptors in DED is largely unexplored. In this study, we use an experimental DED model to investigate the efficacies of novel small molecule Trk agonists. **Pan** and **C1** decreased desiccation-induced corneal barrier disruption while **C1** improved conjunctival goblet cell density. They increase EP4 and TNFAIP3 protein expression and decrease NFkB translocation, suggesting that they can be novel therapies for DED.

## Materials and methods

### Animals

Institutional Animal Care and Use Committees at the Baylor College of Medicine approved all animal experiments (protocol 8510). All studies adhered to the Association for Research in Vision and Ophthalmology for the Use of Animals in Ophthalmic and Vision Research and to the National Institutes of Health guide for the care and use of Laboratory Animals (NIH Publications No. 8023, revised 1978). Experiments were performed at the Ocular Surface Center, Department of Ophthalmology, Baylor College of Medicine (Houston, Texas).

Young breeder pairs of C57BL/6J (B6) mice were purchased from The Jackson Laboratory (Bar Harbor, ME) for establishing colonies. Totally 199 female B6 mice were maintained in a specific pathogen-free vivarium and then used at the age of 6 to 10 weeks. Mice were housed at specific pathogen-free Baylor College of Medicine facilities and were kept on daily cycles of 12 h/light and 12 h/dark with *ad libitum* access to food and water. Dry eye is more frequent in women (5) and aged male mice do not develop corneal barrier disruption (6) (a hallmark of DED), so only females were used. Final sample sizes per endpoint can be found in the Figure legends.

### General procedure for syntheses of **C1** and **pan**, and characterization and evaluation of tyrosine kinase selectivity

*N*-Boc-*cis*-4-*N*-Fmoc-amino-L-proline (Boc is *tert*-butyloxycarbonyl and Fmoc is 9-fluorenyloxycarbonyl; 0.48 mmol) was dissolved in DMF (4 mL) and then DIPEA (di-*iso*-propyl ethyl amine, 1.2 mmol) was added. Half of the solution placed in a syringe with 2-chlorotrityl resin (0.2 mmol) was microwaved at 50*^o^*C for 30 min. The spent solution was drained, and the other half of the reagent solution was added. The mixture was microwaved at 50*^o^*C for 30 min, drained, and the loaded resin was washed by DMF (2 mL) for 3 min. About 20% Piperidine/DMF (2 mL) was added and the mixture was microwaved at 50*^o^*C for 10 min, then the spent solution was drained. The same procedure was repeated once to fully deprotect the Fmoc group. The resin was washed by DMF (2 mL) for three times after each step.

Couplings and deprotection of regular Fmoc-protected amino acids were then implemented on a peptide synthesizer (Liberty Blue, CEM) using a reaction scale of 0.25 mmol. Coupling was performed using Oxyma (activator base, 1.0 M, 1 mL) and DIC (N,N’-Diisopropylcarbodiimide, 0.5 M, 2 mL) activator for the Fmoc-amino acids (0.2 M, 5 mL); resin was mixed and microwaved at 50*^o^*C for 15 min. After this time, the spent solution was drained and the resin was washed with DMF (2 mL) for 3 min. For Fmoc removal, 20% piperidine/DMF (5 mL) was added to the reaction vessel and microwaved at 50*^o^*C for 10 min. The spent solution was drained and the resin was washed by DMF (2 mL) for 3 min.

After repeated coupling and deprotection cycles, the resin-linked linear peptide was transferred back to a syringe. About 20% HFIP/CH_2_Cl_2_ (hexafluoro-*iso*-propanol 3 mL) was added and shaken for 3 h to cleave the peptide from the resin. The solution was stored in a round bottom flask (250 mL), and solvents (HFIP, CH_2_Cl_2_) were removed by airflow. HATU (*N*-[(dimethylamino)-1*H*-1,2,3-triazole-[4,5-*b*]pyridin-1-ylmethyl ene]-*N*-methylmethanaminium hexafluorophosphate *N*-oxide, 0.6 mmol), HOAt (1-Hydroxy-7-azabenzotriazole, 0.6 mmol), 2,4,6-collidine (0.6 mmol), and DMF (60 mL) were added to the flask. The mixture was stirred for 8 h to cyclize the linear peptide. The DMF was removed by high-vacuum rotavapor. Water/acetonitrile (2∼3 mL) was added to dissolve the remaining oils, and the side chain-protected cyclic peptide was purified by preparative HPLC. The purified cyclic peptide is dissolved in 95% TFA/2.5% H_2_O/2.5% TIPS and stirred for 3 h to deprotect all remaining protecting groups. Diethyl ether was added, and the deprotected compound was precipitated and collected after centrifugation for 5 min. The precipitate was dissolved by water/acetonitrile (2∼3 mL). The crude peptide was further purified by preparative HPLC to yield a pure product. Retention times were from analytical HPLC using a Zorbax SB-C18 column (Agilent) with a 20-min gradient between 5% solvent A (99.9% water, 0.1% TFA) and 95% solvent B (99.9% acetonitrile, 0.1% TFA), and 95% solvent A and 5% solvent B. Expected masses were obtained from ESI-MS.

### Cell survival assays

Cells were seeded at a density of 20k per well in 96-well plates in complete media, allowed to adhere for 24 h, then washed with DPBS (Dulbecco’s phosphate-buffered saline) and incubated in serum-free media for 48 to 72 h in the presence of the compound and suboptimal neurotrophin (0.2 nM for NGF and NT-3, and 0.6 nM for BDNF). Cell survival was quantified via AlamarBlue assay and normalized to DMSO (dimethyl sulfoxide 0%) and optimal levels of neurotrophin (100%). A dose response was calculated.

### Storage and preparation of **A1, C1, pan**, and **D3** for *in vitro* and *in vivo* studies

Compounds **A1, C1, pan,** and **D3** (now known as Tavilermide) were kept at –80°C until ready to use. They were dissolved in DMSO to get 20 mM stock and further diluted in either medium for *in vitro* studies or PBS for topical treatment.

### Toxicity assays *in vitro* cornea culture and WST-1 analysis

Primary corneal epithelial cells were established following our publications (7). In brief, C57BL/6J corneas were excised under a dissection microscope and placed in a petri dish with dissecting medium on ice. After trimming off any iris, corneas were cut into four pieces and transferred as explants to a 48-well culture plate. After letting the cornea pieces adhere to the bottom of the well, SHEM medium was added (DMEM + F12 + 10%FBS with EGF 5 ng/ml, cholera toxin A subunit 30 ng/ml, hydrocortisone 21-hemisuccinate sodium salt 0.5 ug/ml, 0.5% DMSO, 1X ITS, gentamicin 50 ug/ml, and amphotericin). Explants were incubated at 37°C with 5% CO_2_. When cornea epithelial cells had grown to almost confluent, **pan** and **C1** were added at 10, 50, and 100 μM, respectively. WST-1 reagent was added at 1:10 according to the company protocol (Abcam, Cat# ab155902). The absorbance of untreated (control) and treated wells was monitored in a microplate reader (OD = 420–480 nm, 0, 2, 4, 6, and 24 h, reference wavelength 650 nm).

### Desiccating stress and topical dosing regimen

Desiccating stress (DS) was induced by inhibiting tear secretion with scopolamine hydrobromide (Greenpark, Houston) in drinking water (0.5 mg/mL) (8) and housing in a cage with a perforated plastic screen on one side to allow airflow from a fan placed 6 inches in front of it for 16 h/day for 1 or 5 consecutive days (DS1, DS5). Room humidity was maintained at 20 to 30%. Control mice were maintained in a non-stressed (NS) environment at 50 to 75% relative humidity without exposure to air draft.

Mice received topical eye drops (5 μl/eye, twice per day) of either with the vehicle (PBS) or increasing concentrations (10 μM, 50 μM, or 100 μM) of **C1**, **A1**, **pan,** or Tavilermide (10 μM). Eye drops were prepared by diluting 20 mM stock concentration into sterile PBS.

### Measurement of corneal barrier function

Corneal barrier function was assessed by quantifying corneal epithelial permeability to 70-kDa Oregon-Green-Dextran-AlexaFluor-488 (OGD; Invitrogen, Carlsbad, CA) according to a previously published protocol but with modifications. In brief, 1 ul of a 50 mg/ml solution of OGD was instilled onto the ocular 1 min before euthanasia. Corneas were rinsed with 2 mL of PBS and photographed with a stereoscopic zoom microscope (model SMZ 1500; Nikon) under fluorescence excitation at 470 nm. OGD staining intensity was graded in digital images by measuring the mean fluorescence intensity within a 2-mm diameter circle placed on the central cornea using NIS Elements software (version AR, 5.20.02) by two masked observers. The mean intensity of the right and left eyes were averaged, and the mean average from biological replicates was calculated and analyzed.

### Histology and periodic acid-schiff staining

Eyes and ocular adnexa were excised, fixed in 10% formalin, paraffin-embedded, and cut into sections (5 mm thick) using a microtome (Microm HM 340E; Thermo Fisher Scientific, Waltham, MA). Sections cut from paraffin-embedded globes were stained with periodic acid Schiff reagent (PAS). Goblet cell densities were measured in the superior and inferior bulbar and tarsal conjunctiva using NIS-Elements software AR, version 5.20.2 (Nikon, Melville, NY), and expressed as a number of positive cells per millimeter.

### NanoString^®^ data analyses using ROSALIND ^®^

A total of 248 transcripts were quantified with the NanoString ^®^ nCounter multiplexed target platform using the mouse Inflammation panel.^1^ Supplementary Table 1 shows the list of all genes in the panel. nCounts of mRNA transcripts were normalized using the geometric means of housekeeping genes (Cltc, Gapdh, GusB, Hprt, were1, and Tubb5). Data were analyzed with a HyperScale architecture developed by ROSALIND ^®^, Inc.^2^ (San Diego, CA). Read distribution percentages, violin plots, identity heatmaps, and sample MDS plots were generated as part of the QC step. Normalization, fold changes, and p-values were calculated using criteria provided by NanoString ^®^. ROSALIND ^®^ follows the nCounter Advanced Analysis protocol of dividing counts within a lane using the same lane’s geometric mean of the normalizer probes. Housekeeping probes for normalization are selected based on the geNorm algorithm implemented in the NormqPCR R library (9). Abundances of cell populations were calculated on ROSALIND ^®^ using the NanoString ^®^ Cell Type Profiling Module. ROSALIND ^®^ performs filtering of cell type profiling to isolate results with a p-value smaller than or equal to 0.05. Fold changes and p-values are calculated using the fast method described in the nSolver ^®^ Advanced Analysis 2.0 User Manual. P-value adjustment is performed using the Benjamini-Hochberg method of estimating false discovery rates (FDR). Clustering genes for the final heatmap of differentially expressed genes was done using the PAM (Partitioning Around Medoids) method using the FPC R library (10) which considers the direction and type of all signals on a pathway for the position, role, and type of every gene. Hypergeometric distribution was used to analyze the enrichment of pathways, gene ontology, domain structure, and other ontologies. The topGO R library was used to determine local similarities and dependencies between GO terms to perform Elim pruning correction. Several database sources were referenced for enrichment analysis, including Interpro, NCBI5, MSigDB (11), REACTOME (12), and WikiPathways (13). Enrichment was calculated relative to a set of background genes relevant to the experiment. Data analyzed in ROSALIND were downloaded and heatmaps were constructed using the GraphPad Prism software.

### Western blotting for TNFAIP3, EP4, and Phospho NFkB p65

C57BL/6J corneal epithelium was scraped and lysed in RIPA lysis buffer (Thermo Fisher, Waltham, MA, Cat# 89900) plus protease inhibitors cocktail (SIGMA, St. Louis, MO, Cat# P8340) or was lysed to extract cytoplasmic and nuclear proteins with a nuclear extraction kit and stored at 80°C until use. A biological sample results from pooling corneas from at least four mice/group. Protein concentration was measured using a micro-BCA protein assay kit (Thermo Fisher, Waltham, MA, Cat# 23235). Cornea and conjunctiva extracts (30 ug) were resuspended in SDS sample buffer, boiled for 5 min, and analyzed on 4 to 15% mini-protean TGX stain-free gels (Bio-Rad, Hercules, CA, Cat# 4568084). The proteins were electrophoretically transferred to polyvinylidene difluoride membranes (Bio-Rad, Cat# 170-4157). Blots were probed with an anti-TNFAIP3 (Table 1), anti-EP4 (Proteintech, Rosemont, IL, Cat# 24895-1-AP), anti-phospho-NFkB p65 antibodies (Abcam, Cambridge, MA, Cat# ab106129), or an anti-actin antibody (SIGMA, St. Louis, MO, Cat# A5441) overnight at 4°C. Blots were washed extensively with a solution containing 50 mM Tris, pH 8.0, 138 mM NaCl, 2.7 mM KCl, and 0.05% Tween 20. Antigen-antibody complexes were detected using the ECL protocol (GE Healthcare, Chicago, IL, Cat# RPN2106) using horseradish peroxidase-conjugated goat anti-mouse IgG as the secondary antibody. Images were taken by ChemiDoc Touch Imaging Systems (Bio-Rad), and band densities were measured by Bio-Rad software (Image Lab version 6.0; Bio-Rad). First, the band intensity of the marker of interest was measured, then we measured the loading control, and then calculated the marker/loading control ratio. Digital images of the whole blots are included in the Supplementary material.

**TABLE 1:** Antibodies used in this study.

Antibody type	Target	Host	Reaction	Conjugation	Company (Catalog No.)	Dilution	Application
Primary	PTGER4	Rabbit	Mouse	none	Proteintech (24895-1-AP)	1:240	IMM
Primary	TNFAIP3	Rabbit	Mouse	none	Proteintech (23456-1-AP)	1:240/1:1000	IMM/WB
Primary	TNFAIP3	Rabbit	Mouse	none	Cell Signaling (5630S)	1:1000	WB
Primary	TNFAIP3	Rabbit	mouse	none	ThermoFisher PA5-86684	1:1000	WB
Primary	pNFκB p65	Rabbit	Mouse	CoraLite^®^594	Proteintech (CL594-10745)	1:40	IMM
Primary	pNFκB p65	Rabbit	Mouse	none	Abcam (ab106129)	1:1000	WB
Primary	PCNA	Mouse	Mouse, Human	none	Abcam (Ab29)		WB
Secondary	IgG (H + L)	Goat	Rabbit	Alexa-Fluor 488^®^	Jackson ImmunoResearch (111-545-003)	1:1000	IMM
Secondary	IgG (H + L)	Goat	Rabbit	HRP	Abcam (ab6721)	1:4000	WB

IMM, immunostaining; WB, western blot.

### Whole-mount immunofluorescence staining and confocal microscopy

Corneas were dissected from female C57BL/6J and fixed in 100% methanol for 20 min at –20°C, followed by washing with HBSS (HANK’s Buffered Sodium Saline) for 3 × 5 min with gentle shaking at room temperature (25°C). Cryosections were prepared using a cryostat and stored at –80°C until ready to use. Corneas or cryosections were permeabilized with 0.4% Triton X-100 in Hanks’s media for 30 min at 25°C and with gentle shaking. Goat serum (20%, Sigma, United States) diluted in HBSS was used for 1 h of blocking at RT. Then, the corneas or eyeball sections were incubated with primary antibodies (Table 1) diluted in 5% goat serum in HBSS with mentioned concentrations overnight at 4°C with gentle shaking at dark. Samples were then washed with 0.4% Triton X-100 for 3 × 6 min at RT with gentle shaking, followed by incubation with secondary antibodies (Table 1) diluted in 5% goat serum/HBSS for 1 h at RT with gentle shaking and light protection. After, the samples were washed for 3 × 10 min with 0.4% Triton X-100 in HBSS and then counterstained with Hoechst (1:500 in HBSS) for nuclei staining (30 min at RT and dark with gentle shaking). Ultimately, the samples were washed 3 × 5 min with HBSS and coverslips were applied. These immunofluorescence stained whole-mount corneas or cryosections were visualized using laser scanning Nikon confocal microscope (Nikon A1 RMP, Nikon, Melville, NY, United States) and 0.5 μm Z-step. Captured images were processed using NIS Elements Advanced Research (AR) software version 4.20 (Nikon). Images were processed using the Denoise function. The intenstiy of staining was calculated by a masked examiner by selecting the function “area autodetect” of NIS Elements, which measures both the intensity and the area. The final intensity was expressed as gray levels/μm^2^ and biological samples within the group were averaged.

### Statistical analysis

Statistical analyses were performed with GraphPad Prism software (GraphPad Software, San Diego, CA, version 9.1). Data were first evaluated for normality with the Kolmogorov–Smirnov normality test. Then, appropriate parametric (*T*-test) or non-parametric (Mann–Whitney) statistical tests were used to compare the two groups. Whenever adequate, one-way or two-way ANOVA or Kruskal–Wallis followed by *post hoc* tests were used. All experiments were repeated at least once. The final sample per experiment is shown in the Figure legends.

## Results

Turn regions in the neurotrophins have a large influence on Trk-selectivity, and mimicry of these has successfully generated small molecules with partial agonistic properties like Tavilermide (1). Crystallographic evidence indicates that NGF binds by burying its three beta-turns (per monomer) into the transmembrane region of TrkA; unfortunately, regions where the turns contact the receptor were not resolved in that structure. Based on this evidence, and from literature studies featuring point mutations and generation of chimeric proteins (1), these turns determine Trk binding selectivities. Based on that hypothesis, Tavilermide was designed by our group to mimic *i* + 1, *i* + 2 residues of the 94, 95 turns in NGF. It proved to be a partial agonist of TrkA and did not bind TrkC or p75. Progression of Tavilermide to phase 3 clinical trials underlines the potential of Trk agonists for the treatment of DED. However, Tavilermide seems to be stalled in phase 3 clinical trials, possibly due to inadequate efficacy. Moreover, it contains an aromatic nitro functionality, which tends to impart poor toxicity profiles. Toxic effects did not emerge in phase 2 trials, but these are relatively short-term, so concerns remain regarding long-term dosage effects for chronic DED. Consequently, we prepared *a new* neurotrophin loop mimicking small molecules, featuring techniques designed and patented by Burgess. New loop mimics were produced to encapsulate amino acids corresponding to key loops in NGF, NT-3, and BDNF (Supplementary Figures 1–3).

Cell survival assays were used to investigate Trk specificities of the new compounds because they provide direct readouts of biological responses. Supplementary Figure 3 shows data for **A1**, **C1**, and **pan**. Stable transfectant cells expressing TrkA to C were used (based on parental cell lines that express none of these receptors or p75; HeLa for A, HEK293 for B, and NIH3T3 for C since they were not able to make them in a single cell line). We tested for cell survival in serum-free media (SFM) as a measure of true agonism (no neurotrophins) and for partial agonism (sub-optimal concentrations of appropriate neurotrophins). One compound, **A1**, was a pure (*i.e.*, not partial) TrkA agonist with selectivity for that receptor over B and C (Supplementary Figure 4). Compound **C1** was a selective partial agonist for C, and **pan** was one of the most potent agonists which activated all three receptors. Dose-response data were recorded for these three compounds (Supplementary Figure 5). Unlike Tavilermide, **A1**, **C1,** and **pan** do *not* contain a nitro group or other functionalities with obvious adverse toxicity liabilities.

Based on these survival data, **A1**, **C1,** and **pan** were tested *in vitro*. Increasing concentrations of **C1** and **pan** were added to confluent murine primary cornea cultures for up to 24 h and toxicities of epithelial cells were evaluated through the WST-1 cell proliferation assay. We observed that both compounds were not toxic to the corneal epithelium (not shown).

### Topical **pan** and **C1** treatment prevents desiccation-induced corneal barrier disruption and goblet cell loss

**FIGURE 1:**
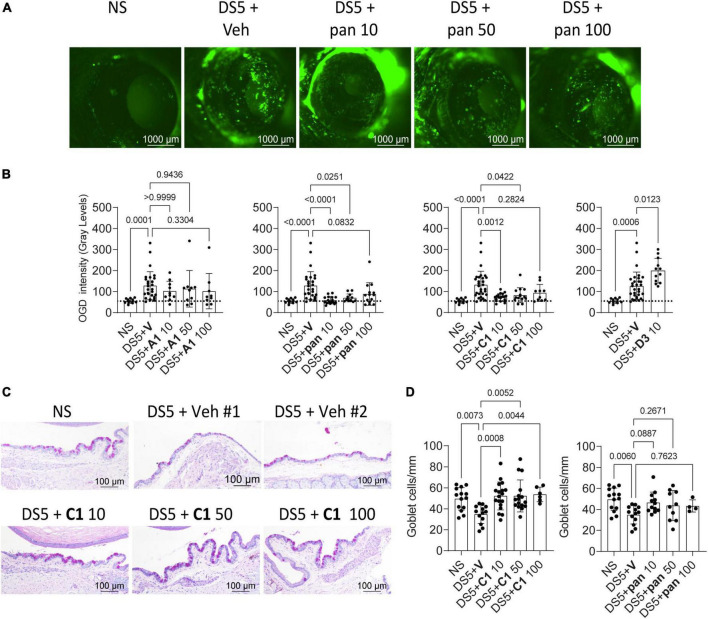
Small molecules improve dry eye disease. **(A)** Representative images of C57BL/6 mice corneas stained with Oregon-green Dextran (OGD) after the mice were subjected to desiccating stress for 5 days (DS5) with topical vehicle or eye drops containing increasing concentrations of pan. Numbers after the compound indicate the concentrations (10, 50, or 100 μM). NS, non-stressed, naïve controls. Scale bar = 1,000 μm. **(B)** Cumulative corneal barrier evaluation in mice subjected to desiccating stress for 5 days (DS5). Mice receiving eye drops of either A1, C1, pan, or D3 (Tavilermide) or vehicle (Veh). Numbers after the compound indicate concentrations (10, 50, or 100 μM). Each dot represents one animal (average of right and left eyes, *n* = 11–31). Non-parametric Kruskal–Wallis non-parametric test followed by Dunn’s comparison. P-Value as shown. **(C)** Representative images of conjunctival sections stained with PAS (purple-magenta) showing increased goblet cell density after topical treatment with C1. **(D)** Cumulative graph of conjunctival goblet cell density. Non-parametric Kruskal–Wallis followed by Dunn’s multiple comparison test. Scale bar = 100 μm. Each dot represents one animal, *n* = 5–18. P-value as shown.

Corneal barrier disruption and goblet cell loss are hallmarks of DED. One of us (CSdP) has published extensively on a desiccating stress model that recapitulates these findings. Thus, the novel Trk analogs were tested in this experimental DED model by subjecting mice to desiccating stress by treating with **A1, pan, C1,** Tavilermide, or vehicle topically, 2x/day *vs.* non-stressed, naïve mice controls. Compared to naïve mice, vehicle-treated, desiccated mice showed corneal barrier disruption, indicating dry eye induction. Dose responses for **A1**, **pan,** and **C1** (10, 50, and 100 μM) were determined while Tavilermide was used at 10 μM. Topical treatment with **A1** and, surprisingly, Tavilermide showed no difference compared to vehicle-treated animals, and investigation of their efficacy was not tested further. Compared to treatment with vehicle, the two smallest doses of **pan** and **C1** (10 μM, 50 μM) showed an improvement in corneal barrier function (Figures 1A,B).

It has been shown that Tavilermide can stimulate mucin production in a rat model of DED (14). Therefore, **pan** and **C1** were tested to see if these would also decrease desiccation-induced goblet cell loss during DS. Eyes and adnexae were collected after 5 days of desiccating stress and histologic sections were prepared and stained with PAS (Figure 1C). Treatment with **C1** significantly increased conjunctival GC density, while treatment with **pan** increased goblet cell density but did not reach statistical significance (Figure 1D).

These results indicate that **pan** and **C1** improve clinical signs of DED but not **A1** nor Tavilermide**. C1** eye drops, but not pan, prevented desiccation-induced goblet cell loss.

### Topical **pan** and **C1** decrease early NFkB translocation in the corneal epithelium

**FIGURE 2:**
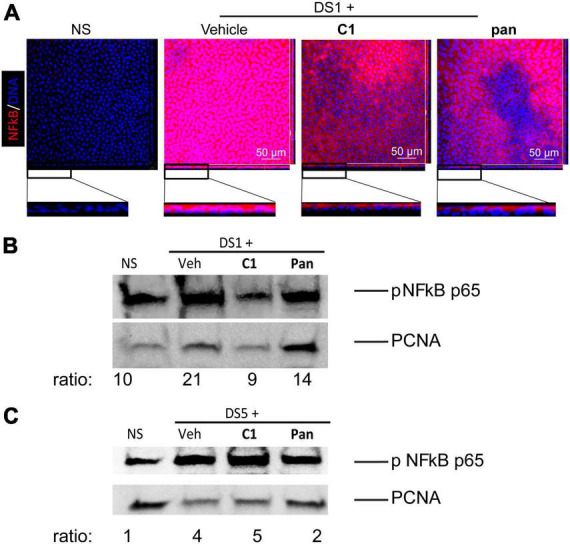
Topical Treatment With C1 and pan Eye Drops Decrease Early Cornea NFkB Nuclear Translocation. Mice were subjected to desiccating stress for either 1 day (DS1) or 5 days (DS5). Whole corneas were used for whole-mount staining, while nuclear corneal epithelial lysates were used for western blot. **(A)** Merged images (en face) of laser confocal microscopy of whole-mount corneas stained with NFkB antibody (red) with DAPI nuclear staining (blue). Insets show images turned at 90 degrees to show individual cells. **(B)** Representative western blot in corneal epithelium lysates of DS1 mice that were blotted with anti-NFkB antibody, or PCNA as a loading control. Each sample contains nuclear proteins obtained from pooling 8 mice (16 corneas) per group. **(C)** Representative western blot in corneal epithelium lysates of DS5 mice that were blotted with anti-NFkB antibody, or PCNA as a loading control. Each sample contains nuclear proteins obtained from pooling 8 mice (16 corneas) per group.

Nuclear factor kappa-light-chain-enhancer of activated B cell (NFkB) has been implicated in autoimmunity, including Sjögren Syndrome. We have previously shown that nuclear translocation of NFkBp65 increased in corneal epithelium after 1 day of desiccating stress (15), so we chose to investigate that timepoint. Mice were subjected to DS for 1 day in experiments to test if our novel agonists (**C1** and **pan**) would prevent this. There was an inverse dose response for our studies evaluating the corneal barrier function, so studies were continued using only the 10 μM dose. Thus, whole corneas were collected for immunostaining and corneal epithelium was scraped and cell nuclear lysates were prepared. Whole-mount corneas were stained with anti-phospho NFkB p65 antibody (Figure 2A). There was a marked cytoplasmic upregulation of NFkB and nuclear translocation in the vehicle-treated corneas after 1 day of desiccation. To our delight, this was diminished by topical treatment with **C1** and **pan** (Figure 2A). Cell lysates were subjected to nuclear extraction, and performed western blot studies to validate our immunostaining results (Figure 2B); both **C1-** and **pan**-treated corneas had decreased band intensities for nuclear phospho-NFkB p65. We also repeated this after 5 days of desiccation: **pan**-treated corneas showed a 50% decrease in NFkB nuclear translocation, but not **C1** (Figure 2C). These observations indicate that both **C1** and **pan** decrease early-desiccation-induced NFkB activation, but only **pan** maintains it until day 5 of desiccating stress.

### Topical treatment with **C1** and **pan** increase anti-inflammatory markers after desiccating stress

Mounting evidence implicates inflammation in DED (16). Anti-inflammatory small molecules have been shown to improve DED (17). Consequently, an unbiased evaluation of genes expressed in corneas treated with **pan** or **C1** (10 μM) was performed and compared to corneas that received vehicle after 5 days of desiccation; each pair was compared individually to the vehicle-treated corneas using the inflammation panel of NanoString (248 genes, Supplementary Table 1). Initially, five biological replicates were processed, but only samples that passed the quality control were further analyzed giving three samples in the vehicle-treated group and four in each treatment group. Analyses were performed using ROSALIND software as described in the methods section.

**FIGURE 3:**
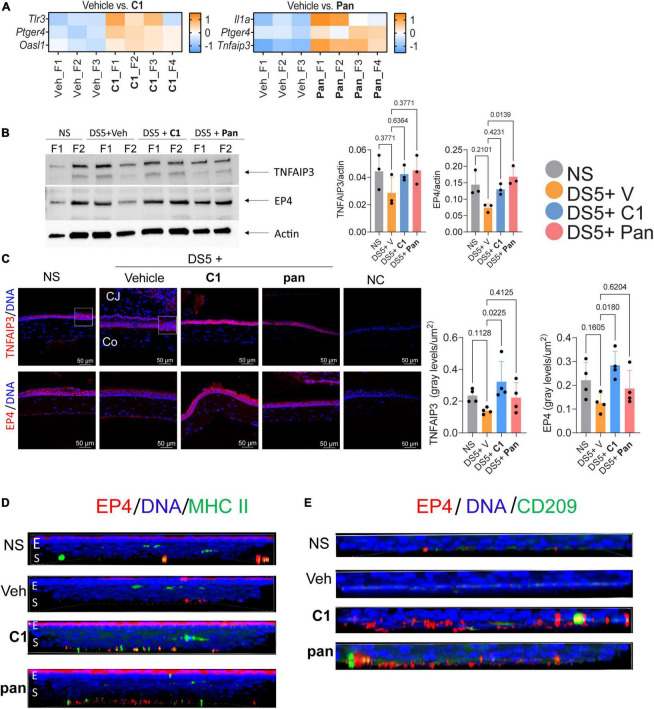
Topical Treatment With C1 and pan Eye Drops Increase the expression of TNFAIP3 and EP4. **(A)** Heatmaps showing differentially expressed genes between the vehicle (Veh) and Pan or C1 corneas. Gene analysis using the NanoString Mouse Inflammation panel v2 was performed on the cornea of mice subjected to desiccating stress for 5 days (*n* = 3–4/group). For all genes identified as significantly changed, log2 > 1.2 and *p*adj. < 0.05. **(B)** Representative western blot in corneal epithelium lysates that were blotted with anti-TNFAIP3 or EP4 antibodies. Beta-actin was used for loading control. Densitometry of the three samples per group is shown in the bar graphs. Non-parametric Kruskal–Wallis followed by Dunn’s multiple comparison test. Each dot represents one biological sample containing cells pooled from four animals. P-value as shown. **(C)** Merged images of laser confocal microscopy of cornea cryosections stained with TNFAIP3 or EP4 antibodies (red) with DAPI nuclear staining (blue). Insets are high magnification to the adjacent area. Bar graphs on the right indicate fluorescence intensity normalized by the area of corneal epithelium. Non-parametric Kruskal–Wallis followed by Dunn’s multiple comparison test. Each dot represents one biological sample. P-value as shown. CJ, conjunctiva; Co, cornea; NC, negative control. **(D)** Merged images of laser confocal microscopy of whole-mount corneas stained with EP4 (red) and MHC II (green) antibodies with DAPI nuclear staining (blue). Z stacks were collected for the EP4 images and rotated 90 degrees to show spatial distribution. E, epithelium; S, stroma. **(E)** Merged images of laser confocal microscopy of the stroma of whole-mount corneas stained with EP4 (red) and CD209 (green) antibodies with DAPI nuclear staining (blue). For EP4 images, Z stacks were collected and images were rotated 90 degrees to show spatial distribution. E, epithelium; S, stroma.

Corneas treated with **pan** showed a significant upregulation of *Il1a*, *Ptger4*, and *Tnfaip3* (encoding TNFAIP3 or A20 protein), and those treated with **C1** showed an increase in *Ptger4*, *Tlr3*, and *Osal1* mRNA transcripts (Figure 3A, log2FC > 1.2, FDR < 0.05). Both *Tfnaip3* and *Ptger4* encode anti-inflammatory proteins.

Experiments were then performed to test if the NanoString results could be validated at the protein level. Expression of prostraglandin E_2_ receptor 4 protein (EP4, encoded by *Ptger4*) was selected for study in more depth because the mRNA was elevated in both treated groups. EP4 is constitutively expressed in the ocular surface epithelium but is absent in severe ocular surface diseases such as Stevens–Johnson Syndrome and Ocular pemphigoid (18, 19). Expression of TNFAIP3 was also tested; expression of this is TNF-induced, and it is involved in the resolution of inflammation by decreasing NFkB. Thus, corneal epithelium was collected after DS5 from all groups and subjected to western blotting. Compared to vehicle-treated animals, treatment with **pan** *or* **C1** increased both TNFAIP3 and EP4 bands; however, only EP4 levels in the **pan**-treated corneas reached statistical significance (Figure 3B). Interestingly, western blot using corneal lysates blotted with anti-TNFAIP3 antibody showed three bands between 150 kDa and 250 kDa instead of the expected 90 kDa. To test if this phenomenon was related to the antibody used in the western blotting, we repeated blotting with two, the ones purchased from two different companies and obtained similar results, indicating that the TNFAIP3 protein has multiple bands and is preferentially found between 150 kDa and 250 kDa in samples from the corneal epithelium.

Next, we performed immunostaining of corneal cryosections stained with TNFAIP3 and EP4 antibodies. Images of the central cornea were obtained with fixed conditions among the groups, and image analysis was used to quantify the intensity. Both TNFAIP3 and EP4 proteins were expressed in the corneal epithelium of naïve mice; TNAIP3 expression was present in membrane-like, while EP4 was more cytoplasmic (Figure 3C). Visible disturbance of TNFAIP3 and EP4 immunoreactivity was observed in vehicle-treated corneas. Treatment with C1 and Pan increased immunoreactivity of TNFAIP3 and EP4 compared to vehicle, but only C1 reached statistical significance (Figure 3C).

Since EP4^+^macrophages^+^ have been reported (20), we collected corneas and performed double-stained with either MHC II or CD209 [as a marker of macrophages (21); Figures 3D,E] antibodies. There were few EP4^+^MHC II^+^ cells in naïve and vehicle-treated corneas (Figure 3D). Very few EP4^+^CD209^+^ cells were present in the naïve and vehicle-treated corneas, but there were easily identified in the stroma of **C1**- and **pan**-treated corneas.

Together, these results indicate that both **C1** and **pan** increase the anti-inflammatory TNFAIP3 and EP4 mRNAs and there is an influx of EP4^+^ macrophages after treatment.

## Discussion

Dry eye is one of the most, possibly the most, prevalent diseases in the United States; it has significant economic impacts on individuals and societies. Since there are only four FDA drugs that have been approved for DED, there is an unmet need to find new therapeutics. In the past, we synthesized another neurotrophin analog termed tavilermide (22). Studies in a rat model of DED showed that tavilermide was efficacious in decreasing corneal staining scores compared to vehicle and it also promoted glycoprotein secretion (14). This compound was later developed by Mimetogen and it was in clinical trials for DED. Moreover, recombinant human NGF is currently in Phase 3 trials (clinicaltrials.gov, studies NCT05133180; NCT05136170) for the same condition.

Here, the new neurotrophin small molecules with selective binding to TrkA (**A1**), TrkC (**C1**), and a compound with pan-Trk selectivity (**pan**) were tested *in vivo* in an experimental model of DED. Compounds were not cytotoxic using transfectants and wild-type HeLa, NIH3T3, and HEK cell lines; in fact, they promoted the survival of the cell lines transfected with the Trk receptors, but not in the wild-type lines, with Trk specificity and dose response. These two features, lack of cytotoxicity and selective response to only the stable transfectants and not the wild-type cells, speak to the specificity of these interactions. Using primary corneal epithelial cells, we did not observe any toxicity to corneal epithelial cells. Significant improvement in clinical signs was observed accompanied by upregulation of the anti-inflammatory genes *Ptger4* and *Tnfaip3* (confirmed in studies of protein expression).

Surprisingly, the pure, TrkA-selective, agonist **A1** was not efficacious in the DED model, and neither was the preclinical partial TrkA agonist Tavilermide. Evidence shows that NGF is beneficial in wound healing in the cornea and DED, and NGF promotes healing of corneal ulcers (23). These healing roles of NGF seem to be related to its ability to increase the proliferation of corneal epithelial cells, in a similar way that this growth factor in the skin stimulates growth around a wound margin (24). Furthermore, treatment with NGF increases goblet cell density and production of Muc5ac *in vivo* and in cultures (25). A human recombinant NGF (Cenergermin) has been FDA-approved for neurotrophic keratitis and it is currently under clinical trial for severe Sjögren-Syndrome DED. Thus, it is surprising that **A1** and Tavilermide were *not* efficacious in the DED model (at the concentrations and dosing regime used), whereas compounds that have TrkC selectivity and broad Trk selectivity were efficacious (**C1** and **pan**, respectively). These were efficacious in ameliorating cornea (**C1, pan**) and conjunctival disease (just **C1**) by improving corneal barrier function and goblet cell densities. Cornea disease is a hallmark of all different types of DED and goblet cell loss is characteristic of aqueous-tear deficient DED. Cornea disease leads to cornea irregularity, blurred vision, and foreign body sensation while goblet cell loss deprives the ocular surface of immunoregulatory factors such as mucins, TGF-β, and retinoic acid (26).

Our results showed that both **C1** and **pan** decreased early nuclear translocation of NFkB. The involvement of NFkB in DED and Sjögren Syndrome is well-established in the literature (15, 27). Indeed, constitutive transgenic expression of NFkB in acinar cells is sufficient to induce the Sjögren syndrome phenotype in mice (28). Our results also showed that while only **pan** eye drops increased the *Tfnaip3* mRNA, both small molecules increased TNFAIP3 protein expression in the cornea after desiccation stress. However, only **C1**-treated corneas showed statistical significance in immunostained corneas. It is possible that with larger sample size, **pan**-treated corneas would have also reached statistical significance (although it did so in the cell lysates subjected to western blot). TNFAIP3 (or A20) is a negative regulator of NFkB and polymorphisms in the *TFNAIP3* gene, which has been associated with autoimmune diseases, including Sjögren Syndrome (29). Epithelial Keratin 14-specific deletion of TNFAIP3 induces a Sjögren Syndrome-like in mice, recapitulating immune infiltration and a decrease in saliva production (27). TNFAIP3 also negatively regulates CCL2, CCL5, and IL12A (30) markers that have been implicated in DED.

Our studies showed that **C1** and **pan** eye drops increased *Ptger4* mRNA and the corresponding protein expression in the cornea after desiccating stress reaching statistical significance for **C1** in immunostained corneas. It is possible that with larger sample size, it might also reach statistical significance with **pan**. Observation of immune cells was positive for EP4 protein, and mostly macrophages support the involvement of this protein in the healing effect. EP4 expression in the normal cornea has been previously described (19). Downregulation of EP4 occurs in severe ocular surface conditions such as Stevens-Johnson, graft-versus-host-disease (18). Our studies agree with the literature indicating that EP4^+^ macrophages promote epithelial barrier repair in a dextran sulfate sodium-induced colitis model (20). Furthermore, EP4 expression in macrophages is needed for their anti-inflammatory response *in vitro* (20).

Taken together, our results indicate that novel Trk agonists are protective during desiccating stress by decreasing early NFkB nuclear translocation and upregulating epithelial and macrophage-related anti-inflammatory markers. Surprisingly, the most efficacious small molecule agonists were not TrkA selective, but TrkC and panTrk, suggesting that wider exploration of TrkB and C and pan Trk agonists are warranted in efforts to DED.

## Data availability statement

The original contributions presented in this study are included in the article/Supplementary material. Uncropped western blot images are provided as Supplementary Figures 6–12. Further inquiries can be directed to the corresponding author. The datasets for this study can be found in the GEO repository (GSE202378).

## Ethics statement

The animal study was reviewed and approved by Institutional Animal Care and Use Committees at Baylor College of Medicine.

## Author contributions

KB and CP contributed to the conceptualization, methodology, and funding acquisition. CP and ZY performed and analyzed the *in vivo* data and writing—original draft. SJ performed the cell survival data and reproduced the synthesis of Tavilermide. KB contributed to the design of **A1, C1**, and **pan** with suggestions from TM. CP contributed to the project administration and supervision. CP, KB, ZY, SJ, and TM contributed to the writing—review and editing. All authors contributed to the article and approved the submitted version.

## Footnotes

1
www.nanostring.com

2
https://rosalind.onramp.bio/
