# Supplementary material for: New, potent, small molecule agonists of tyrosine kinase receptors attenuate dry eye disease
Source: Front Med (Lausanne). 2022 Aug 25;9:937142. doi: 10.3389/fmed.2022.937142 (PMC9453230; doi:10.3389/fmed.2022.937142)
Supplement: Supplementary file 1 [file Data_Sheet_1.PDF]

New, Potent, Small Molecule Agonists of Tyrosine Kinase Receptors Attenuate Dry Eye Disease

Zhiyuan Yu[1], Shaon Joy[2,] Tianxiong Mi[2,] Ghasem Yazdanpanah[1], Tye S Thompson[2], Kevin Burgess[2], Cintia S. de Paiva[1]

[1]Ocular Surface Center, Department of Ophthalmology, Cullen Eye Institute, Baylor College of Medicine, Houston, Texas

[2]Department of Chemistry, Texas A&M University, Box 30012, College Station, TX 77842, USA.

CORRESPONDING AUTHOR: Cintia S. de Paiva
Ocular Surface Center
Cullen Eye Institute, Baylor College of Medicine
6565 Fannin St., NC505, Houston, Texas 77030
Phone: 713-798-2124, Fax: 713-798-1457
email: cintiadp@bcm.edu

Reaction Scheme

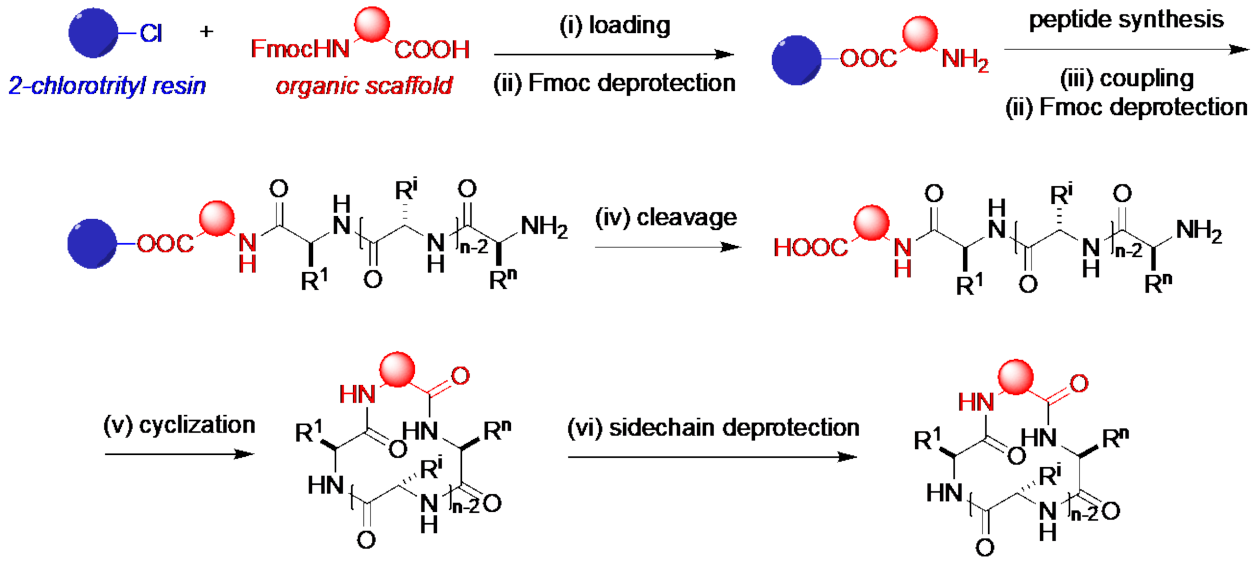

Supplemental Fig. 1. Syntheses of **A1**, **C1**, and **pan**. Conditions:
(i) loading: *N,N*-Diisopropylethylamine (DIPEA), dimethylformamide (DMF), 50 °C, 30 min, microwave; (ii) Fmoc deprotection: 20% piperidine/DMF, 50 °C, 10 min, microwave; (iii) coupling: Oxyma, *N,N'*-Diisopropylcarbodiimide, 50 °C, 15 min, microwave; (iv) cleavage: 20% hexafluoroisopropanol/dichloromethane, 25 °C, 2 h; (v) cyclization: HATU, HOAt, 2,4,6-collidine, DMF, 25 °C, 8h; (vi) sidechain deprotection: 95% trifluoroacetic acid (TFA), 2.5% H_2O, 2.5% triisopropylsilane, 25 °C, 2h.

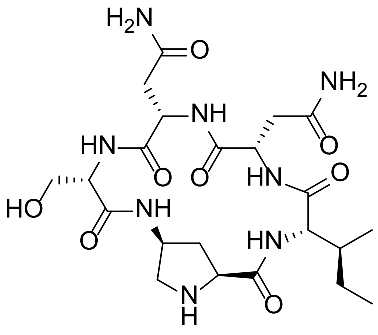

A1

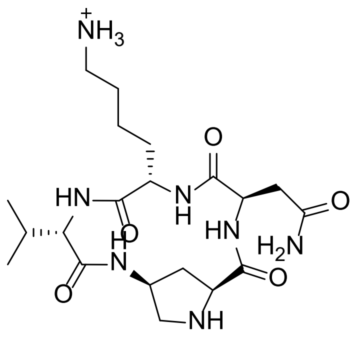

C1

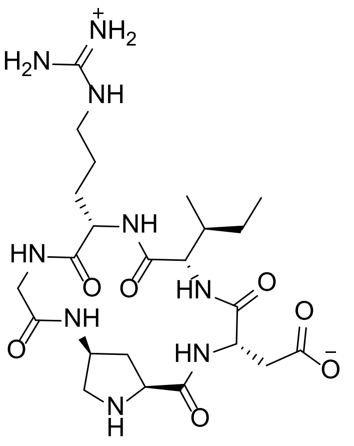

pan

<u>Supplemental Fig. 2. Chemical structures of A1, C1 and pan</u>.

Characterization Data

A1

HPLC

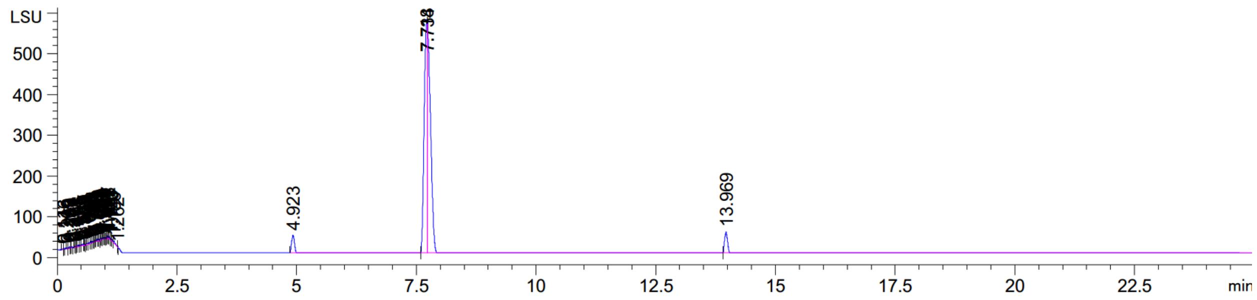

MS

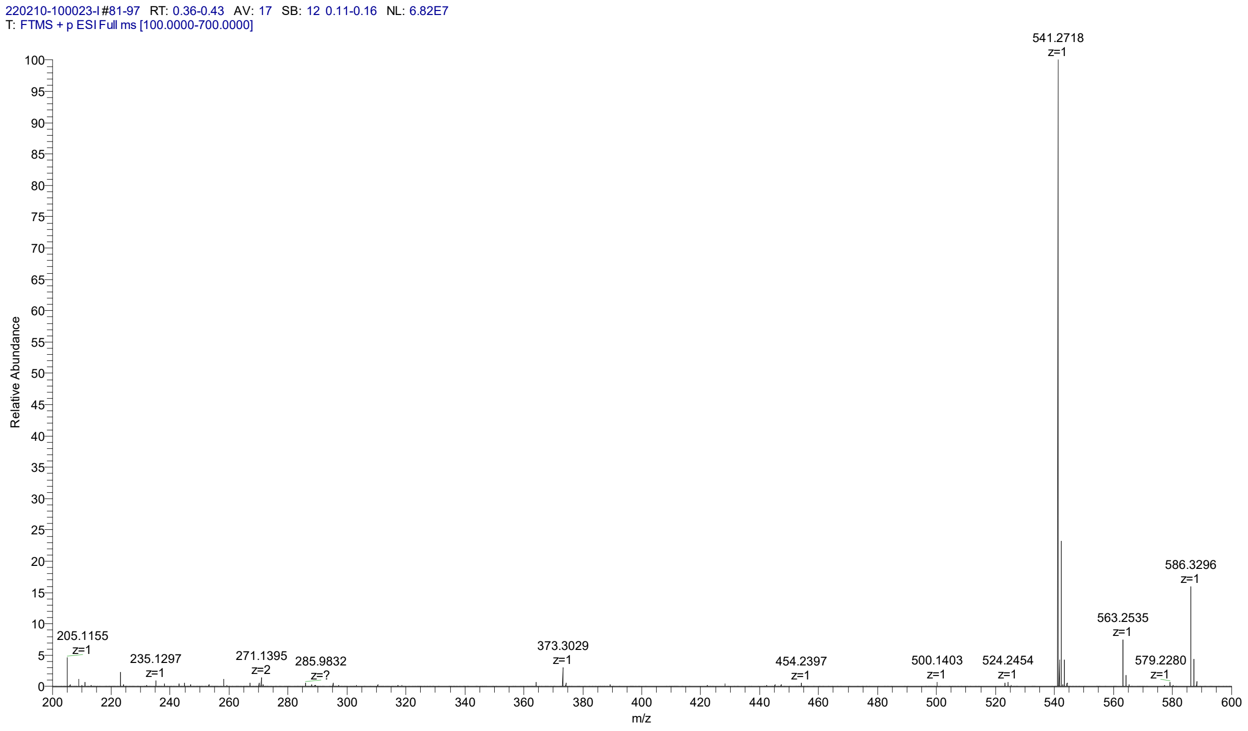

C1

HPLC

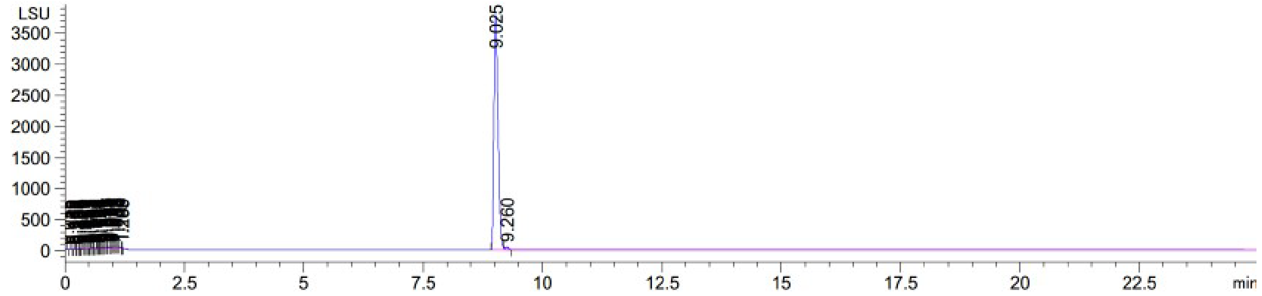
LSU
3500
3000
2500
2000
1500
1000
500
0
9.025
9.260
0
2.5
5
7.5
10
12.5
15
17.5
20
22.5
min

MS

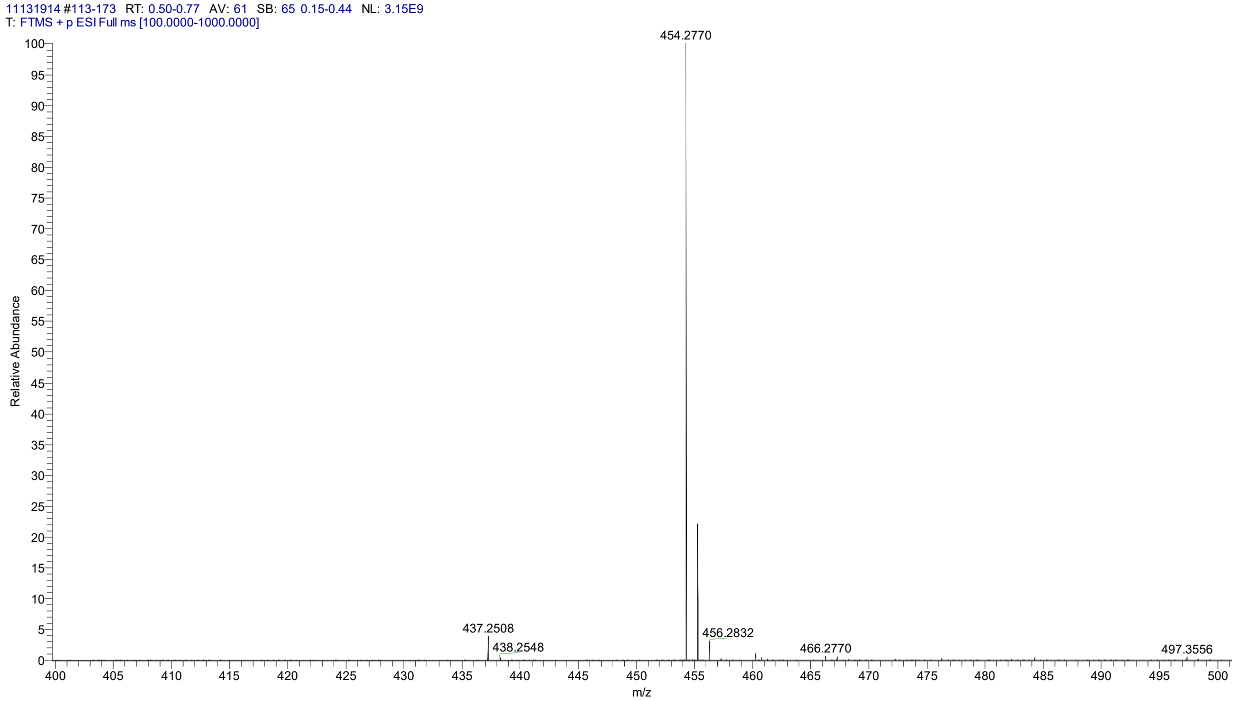
11131914 #113-173 RT: 0.50-0.77 AV: 61 SB: 65 0.15-0.44 NL: 3.15E9
T: FTMS + p ESI Full ms [100.0000-1000.0000]
454.2770
437.2508
438.2548
456.2832
466.2770
497.3556
Relative Abundance
m/z

Pan

HPLC

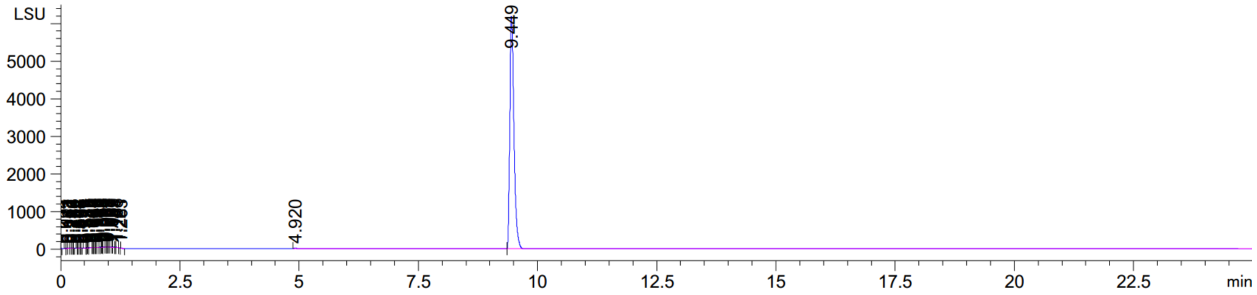

MS

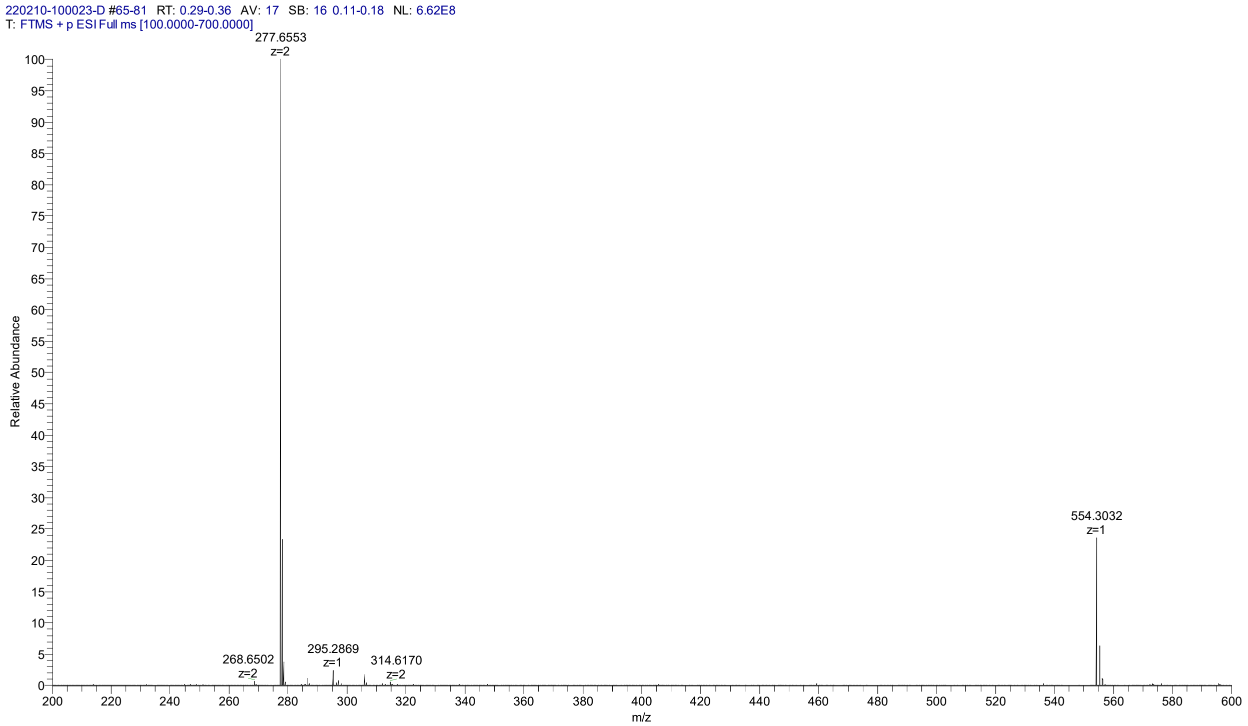

Supplemental Figure 3
Characterization of **A1**, **C1**, and **pan**. High-Performance Liquid Chromatography (HPLC) runs were to show the purity of compounds. Each compound was represented by one peak and its amount is related to the intensity of the peak. All compounds are at least 90% pure from the HPLC traces shown here. Mass spectra (MS) data from electrospray ionization (ESI) showed the $(M+H)^{+}$ *and/or* $(M+2H)^{2+}$ peaks. By comparing observed peaks with expected ones from the calculation, all of the compounds here are correct and pure.

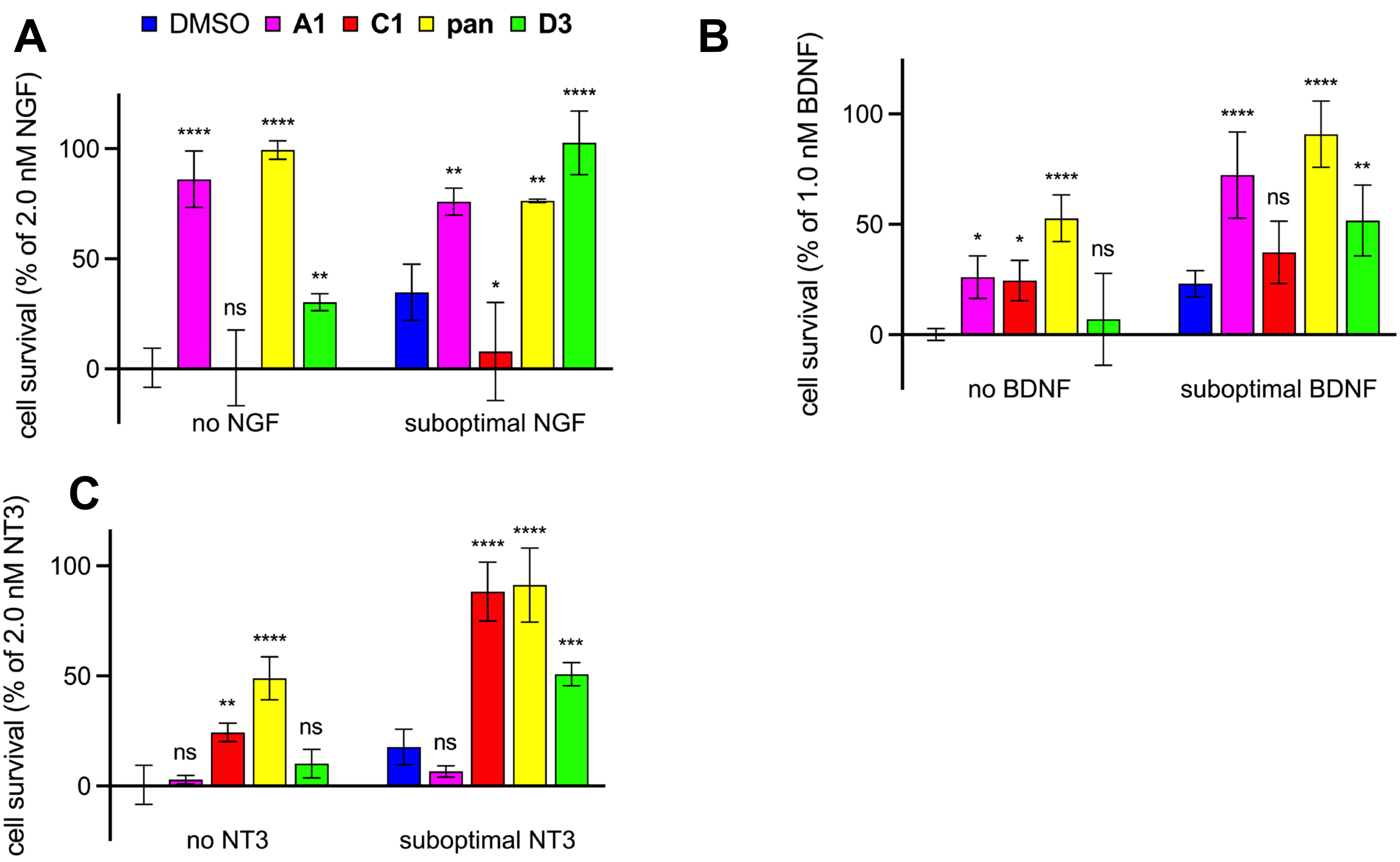

Supplemental Figure 4

Trk-selectivity comparison of each compound at 50 uM in HeLa-TrkA (A) and 0.4 uM in HEK293-TrkB (B) or at NIH3T3-TrkC (C). Compounds were tested with and without suboptimal neurotrophin (0.2 nM NGF or NT3, 0.6 nM BDNF) to differentiate true agonists from partial agonists. Data were normalized to DMSO control and maximum survival imparted by neurotrophin. Data analysis was conducted via two-way ANOVA followed by Dunnett's t-test (compared to DMSO): *$P < 0.05$, **$P < 0.01$, ***$P < 0.001$, ****$P < 0.0001$.

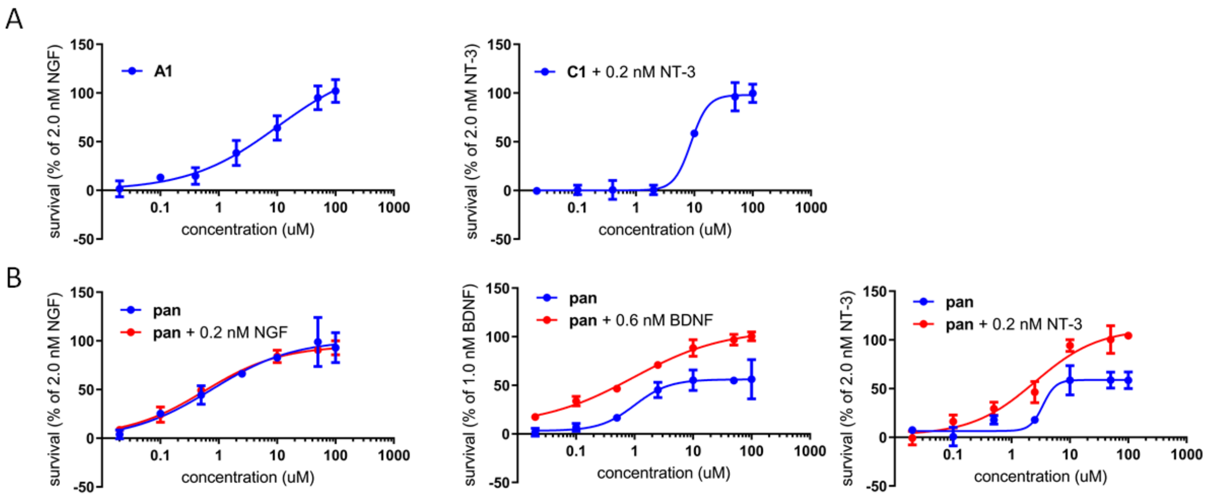

Supplemental Figure 5

Neuronal survival dose-response curves of **A1**, **C1** and **pan** with or without suboptimal concentration of neurotrophins. (A) Cell survival as a function of compound concentration in HeLa-TrkA (left) and NIH3T3-TrkC (right). (B) Cell survival as a function of compound concentration in HeLa-TrkA (left), HEK293-TrkB (middle), and NIH3T3-TrkC (right). In each case, the survival was normalized to the survival achieved either with 2.0 nM NGF or 1.0 nM BDNF or 2.0 nM NT-3. Survival values for each concentration from n=4 wells derived from 3 different experiments were analyzed.

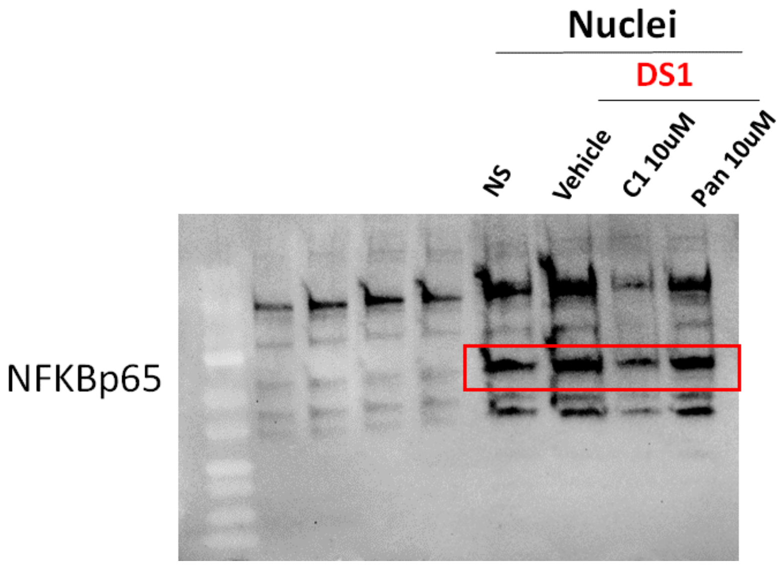

Supplemental Figure 6. Uncropped western blot of nuclear cell lysates of the experimental groups blotted with NFkB p65 antibody (relates to Fig. 2B). Mice were subjected to desiccating stress for 1 day (DS1). Nuclear corneal epithelial lysates were used for western blot.

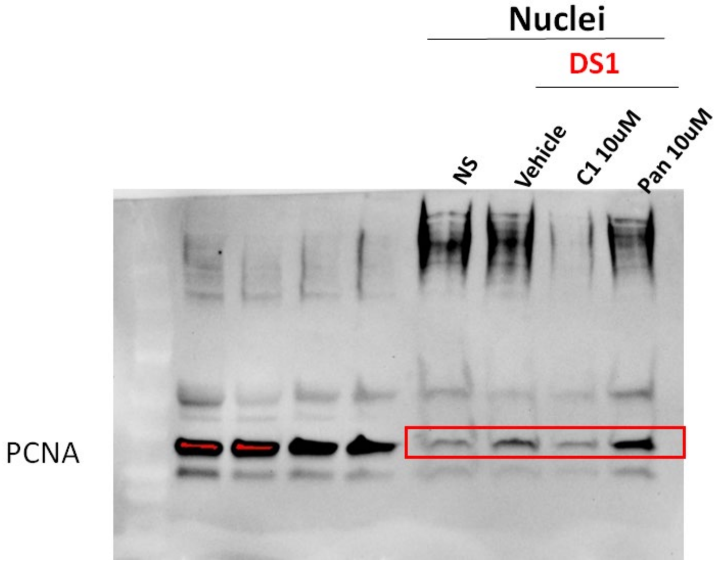

Supplemental Figure 7. Uncropped western blot of nuclear cell lysates of the experimental groups blotted with PCNA (relates to Fig. 2B). Mice were subjected to desiccating stress for 1 day (DS1). Nuclear corneal epithelial lysates were used for western blot.

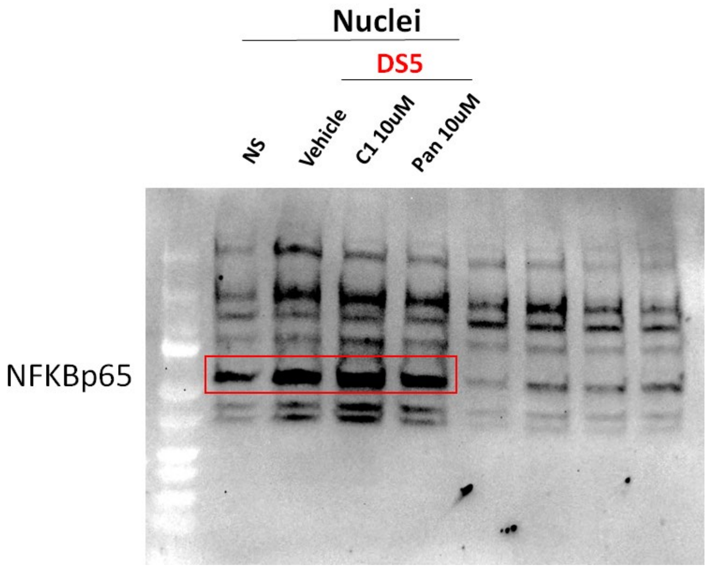

Supplemental Figure 8. Uncropped western blot of nuclear cell lysates of the experimental groups blotted with NFkB (relates to Fig 2C). Mice were subjected to desiccating stress for 5 days (DS5). Nuclear corneal epithelial lysates were used for western blot.

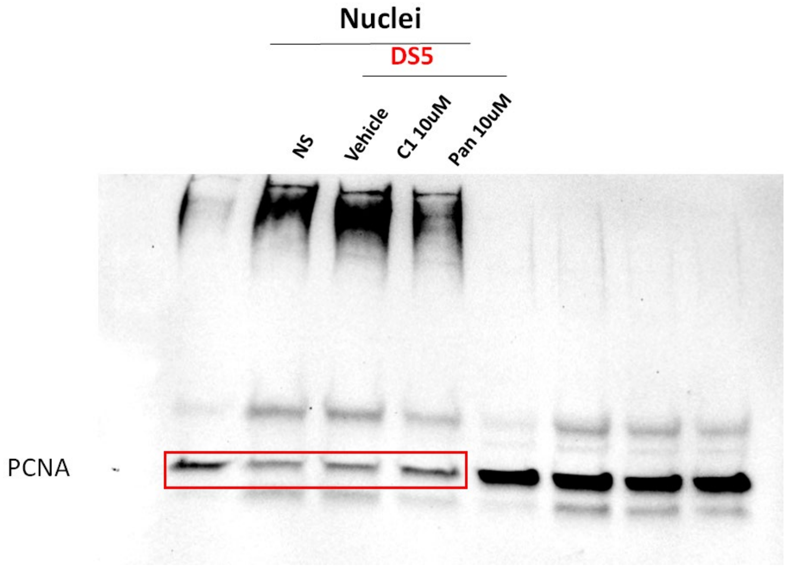

Supplemental Figure 9. Uncropped western blot of nuclear cell lysates of the experimental groups blotted with PCNA (relates to Fig 2C). Mice were subjected to desiccating stress for 5 days (DS5). Nuclear corneal epithelial lysates were used for western blot.

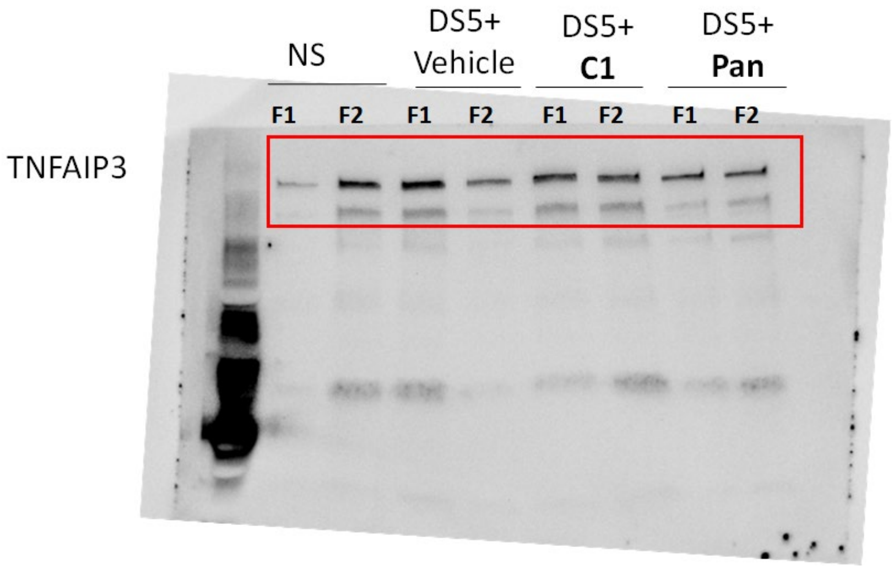

Supplemental Figure 10. Uncropped western blot of total cell lysates of the experimental groups blotted with TNFAIP3 (relates to Fig. 3B). Mice were subjected to desiccating stress for 5 days (DS5). Total corneal epithelial lysates were used for western blot.

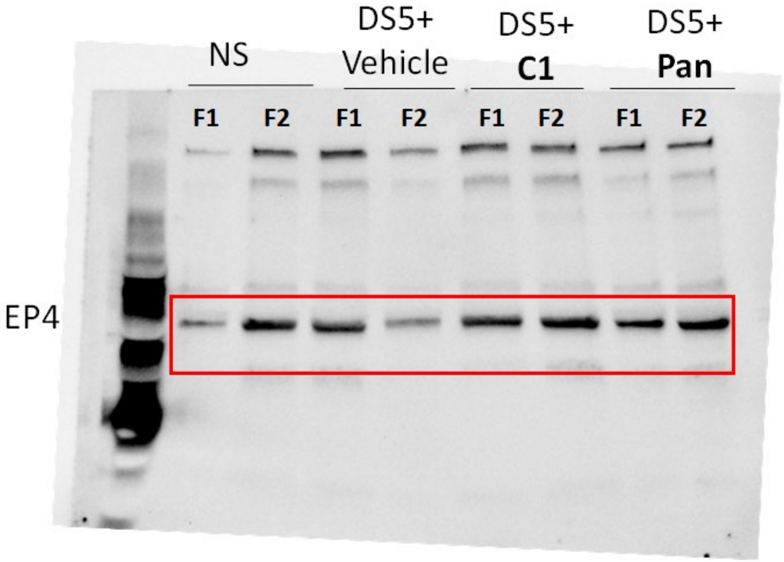

Supplemental Figure 11. Uncropped western blot of total cell lysates of the experimental groups blotted with EP4 (relates to Fig 3B). Mice were subjected to desiccating stress for 5 days (DS5). Total corneal epithelial lysates were used for western blot

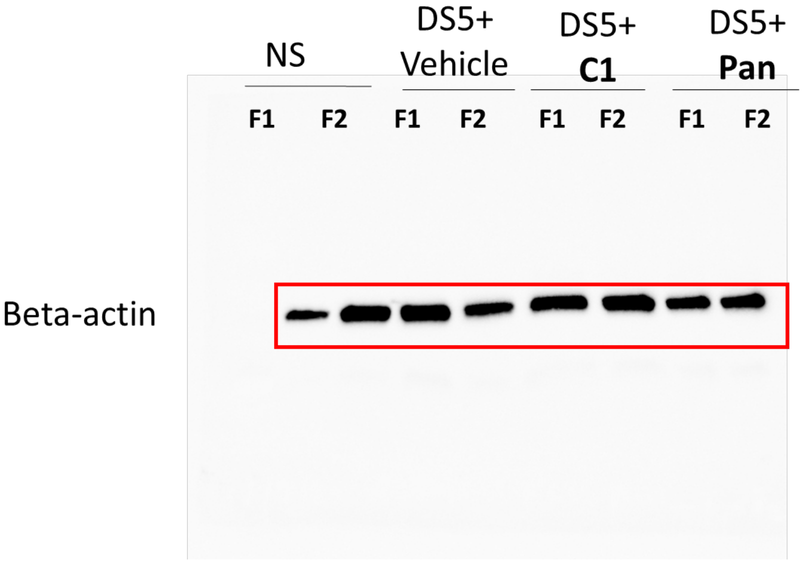

Supplemental Figure 12. Uncropped western blot of total cell lysates of the experimental groups blotted with beta-actin (relates to Fig 3B). Mice were subjected to desiccating stress for 5 days (DS5). Total corneal epithelial lysates were used for western blot.
